# Decompression Mechanism of Radish Seed in Prehypertension Rats through Integration of Transcriptomics and Metabolomics Methods

**DOI:** 10.1155/2023/2139634

**Published:** 2023-01-31

**Authors:** Qiang Jia, Yuchen Qi, Hanbo Li, Hai Ding, Dongmei Qi, Yunlun Li

**Affiliations:** ^1^College of Pharmacy, Shandong University of Traditional Chinese Medicine, Jinan 250355, China; ^2^Innovative Institute of Chinese Medicine and Pharmacy, Shandong University of Traditional Chinese Medicine, Jinan, Jinan 250355, China; ^3^First Faculty of Clinical Medicine, Shandong University of Traditional Chinese Medicine, Jinan 250355, China; ^4^Department of Peripheral Vascular Disease, Affiliated Hospital of Shandong University of Traditional Chinese Medicine, Jinan 250011, China; ^5^Faculty of Traditional Chinese Medicine, Shandong University of Traditional Chinese Medicine, Jinan 250355, China; ^6^Experimental Centre, Shandong University of Traditional Chinese Medicine, Jinan 250355, China; ^7^Department of Cardiology, Affiliated Hospital of Shandong University of Traditional Chinese Medicine, Jinan 250011, China

## Abstract

Radish seed (RS), the dried ripe seed of *Raphanus sativus* L., is widely used in traditional Chinese medicine (TCM) to reduce blood pressure. However, the molecular and pharmacological mechanisms underlying its therapeutic effects are still unclear. In this study, we analyzed the effects of RS in a rat model of prehypertension and assessed the mechanistic basis by integrating transcriptomics and metabolomics. RS administration significantly reduced blood pressure in prehypertensive male Wistar rats, negatively regulated endothelin-1, increased nitric oxide levels, and reduced the exfoliation of endothelium cells. In vitro vascular ring experiments further confirmed the effects of RS on vascular endothelial cells. Furthermore, we identified 65 differentially expressed genes (DEGs; *P*_adj_ < 0.05 and fold change (FC) > 2) and 52 metabolites (VIP > 1, *P* < 0.05 and FC ≥ 2 or ≤0.5) in the RS intervention group using RNA-seq and UPLC-MS/MS, respectively. A network of the DEGs and the metabolites was constructed,q which indicated that RS regulates purine metabolism, linoleic acid metabolism, arachidonic acid metabolism, circadian rhythm, and phosphatidylinositol signaling pathway, and its target genes are Pik3c2a, Hspa8, Dnaja1, Arntl, Ugt1a1, Dbp, Rasd1, and Aldh1a3. Thus, the antihypertensive effects of RS can be attributed to its ability to improve vascular endothelial dysfunction by targeting multiple genes and pathways. Our findings provide new insights into the pathological mechanisms underlying prehypertension, along with novel targets for the prevention and treatment of hypertension.

## 1. Introduction

Prehypertension has been defined by the 2020 Global Hypertension Practice Guidelines as blood pressure (BP) in the range of 130–139/85–89 mmHg [1]. Prehypertensive patients have a greater risk of developing myocardial infarction, incident stroke, and cardiovascular disease (CVD) due to pathological changes in the blood vessels and humoral nervous system [2]. The renal renin-angiotensin system, sympathetic tone [3], immune cell infiltration [4], and endothelial dysfunction (ED) play an important role in the pathogenesis of hypertension [5]. ED is characterized by elevated endothelin (ET)-1 and decreased NO bioavailability, which impairs vascular reactivity [6]. ET-1-mediated vasoconstriction is increased in prehypertensive adults, and its activity may increase with age [7]. Furthermore, prehypertension is associated with impaired NO-mediatedendothelium-dependent vasodilation [8], and NO supplementation can lower BP in prehypertensive individuals [9]. Since prehypertension can progress to hypertension and increase the risk of cardiovascular events and death [10], pharmacological intervention is recommended in prehypertensive individuals [11].

Radish seed (RS), the dried ripe seed of *Raphanus sativus* L., has been used as food and medicine for thousands of years in China. It is prescribed for the treatment of peptic diseases, respiratory diseases, hypertension, and cardiac diseases [12, 13]. We identified its main active compounds, sinapine thiocyanate and glucoraphanin [14, 15], which had a good effect on the regulation of blood pressure [16, 17]. RS extract mediates antihypertensive and anti-inflammatory effects [18]. The curative effect of RS against prehypertension and the pharmacological mechanisms are not completely clear, which limits its clinical applications.

Transcriptomics and metabolomics are increasingly being used to study the mechanisms of various drugs [19–22]. Transcriptomics refers to the complete set of RNA transcripts, including messenger, ribosome, and noncoding RNAs, that are produced by cells or organisms under specific conditions and reflects the gene expression profile associated with physiological and pathological conditions. In recent years, it has been used to identify markers of hypertension-related diseases [23, 24]. Metabolomics is the quantitative analysis of metabolites within cells, tissues, and organisms and is used to identify metabolic changes associated with different physiological and pathological states, as well as drug interventions [25, 26]. This approach has been used to elucidate the pharmacological mechanisms of traditional Chinese medicine (TCM) formulations [17, 27]. While transcriptomics provides genotypic information, metabolomics represents the actual phenotype of the organism since metabolites are the final results of the transcriptional program. Therefore, the integration of transcriptomics and metabolomics can elucidate the complex molecular mechanisms and regulatory networks underlying disease progression and drug action, while obviating the unreliability of single omics sequencing. In this study, we integrated metabolomics and transcriptomics to determine the relationship between differentially expressed genes (DEGs) and differential metabolites (METs) associated with RS intervention in prehypertension in order to identify potential pharmacological mechanisms.

## 2. Materials and Methods

### 2.1. Chemicals and Drugs

Mass spectrometry-grade methanol, acetonitrile, and formic acid were acquired from Thermo Fisher Scientific Inc. (Loughborough, United Kingdom). RS was purchased from Jinan Jianlian TCM Pharmacy (Batch No. 210415, Shandong, China) and authenticated and conformed to the standards of the Pharmacopoeia of the People's Republic of China. To prepare the RS extract, 3 kg of RS was reconstituted with 60 liters of water and extracted twice. The extraction yield was 13.4% (g/g). Details of the three major compounds in the RS extract are shown in Table S1 and Figure S1 in the Supplementary Material. The extract was dried in an oven and dissolved in distilled water to a final concentration of 1 g/ml. Valsartan (Lot X2882, Beijing Novartis Pharma Ltd., China) was also dissolved in distilled water to a final concentration of 1.44 mg/ml. N′-nitro-L-arginine (L-NNA) (Lot BCCD9665) was obtained from Sigma Aldrich (St. Louis, MO, United States), and N^G^-Nitro-L-arginine Methyl Este (L-NAME) (Lot N1101A) was purchased from Meilunbio (Dalian, Liaoning, China).

### 2.2. Animals

Fifty healthy male Wistar rats (body weight: 150–180 g) were purchased from Vital River Laboratory Animal Technology Co., Ltd., Beijing, China (Animal Qualification Certificate No. SCXK (Jing) 2016-0006). All animal experiments were approved by the Animal Care and Ethics Committee of Shandong University of Traditional Chinese Medicine (No. YYLW2021000015). The animals were housed in a standard laboratory environment (temperature 22 ± 2°C, humidity 55 ± 5%, and 12-h light/dark cycle) and provided water and food ad libitum. Prehypertension was modelled by injecting the rats daily intraperitoneally with L-NNA (7.625 mg/kg) twice a week for three weeks [18], whereas the control group (C) received the same volume of physiological saline. The BP was measured after injections to confirm prehypertension (systolic BP is approximately 160 mmHg). The successfully modelled animals were randomly divided into untreated prehypertension (PHT), RS-treated (*R*), and valsartan-treated (*V*) groups, and the respective saline doses of 2.5 g/kg/day for RS and 14.4 mg/kg/day for valsartan were given via the intragastric route for six weeks.

### 2.3. BP Measurements

Arterial systolic and diastolic BP (SBP and DBP; mmHg) of the rats were monitored once a week at the same time each day during the modelling and drug intervention periods using the BP-2000 Blood Pressure Analysis System (Visitech Systems, Inc., North Carolina, USA) with the noninvasive tail‐cuff method. Briefly, the rats were allowed to move inside a holder for 10 min to ensure good blood flow to the tails, and five preliminary measurements were taken in each session. The room was kept free of noise or disturbance to increase the reliability and reproducibility of BP measurements. All rats were examined six times in parallel.

### 2.4. In Vitro Vascular Ring Model

Rats were anesthetized with pentobarbital (50 mg/kg, i.p.) and placed supine on the operating surface.

The thoracic aorta was quickly dissected and placed in an ice-cold Krebs solution that had been prepared 20 min in advance under 95% O_2_ and 5% CO_2_. The surrounding fat and connective tissue were carefully removed from the aortas under a dissecting microscope while avoiding traction and injury to blood vessels, and 3 mm long vascular rings with intact endothelium were mounted in an Multiwire Myograph System (DMT 620M, Myo Technology A/S, Danish), bathed in 5 ml Krebs solution at 37°C, and blistered continuously with mixed gas. The vessels were then stretched in a stepwise manner to their optimal resting tension of 20 mN and then balanced for 1 h before the experiment. The thoracic aortic segment was excited twice with a 5 ml, 60 mM potassium (K^+^) solution to test the functional integrity of the vascular smooth muscle. 1 *μ*M phenylephrine (PE) was added to preconstrict the vascular ring, followed by 10 *μ*M acetylcholine (ACh). The vascular endothelial function was considered intact if blood vessels relaxed to more than 80%. The chambers were then washed until the vascular ring tension returned to the baseline level. In addition, the aortas rings were incubated with L-NAME for 20 min and then treated with PE and RS. The tension change was recorded using the PowerLab 8 signal analysis system. The effect of the different interventions on the vascular ring was calculated in terms of the maximum diastolic rate, that is, the percentage of contraction after and before administration.

### 2.5. Sample Acquisition and Histological Assay

The animals were deeply anesthetized with pentobarbital, and blood was collected by abdominal aorta puncture and centrifuged at 3500 rpm for 15 min to obtain serum. The thoracic aorta was immediately dissected, and one part was immersed in 2.5% glutaraldehyde (Lot: CR2101167, Cusabio, China) and one in 4% paraformaldehyde (Lot: CR2101179, Cusabio, China) for scanning electron microscopy (SEM) and hematoxylin and eosin (HE) staining, respectively. In addition, a small portion was snap frozen for molecular analyses.

### 2.6. Measurement of Endothelin-1 (ET-1) and NO

The levels of ET-1 and NO in the rat sera were measured using a specific Rat Endothelin-1 Enzyme-Linked Immunosorbent Assay Kit (Lot: G14012112, Wuhan Cusabio Co., Ltd., China) and Nitric Oxide Assay Kit (Lot: 20210604, Nanjing Jiancheng Bioengineering Institute, China) as per the manufacturer's instructions.

### 2.7. RNA Sequencing

Total RNA was extracted from the frozen aorta using TRIzol reagent (Lot: 252612, Thermo Fisher Scientific Inc., USA) according to the manufacturer's instructions. Samples were subjected to agarose gel electrophoresis to detect any contamination or degradation, and the purity and concentration of RNA were determined using the NanoPhotometer®N120 spectrophotometer (IMPLEN Corp., Munich, Germany). The integrity and quantity of RNA were assessed by the Agilent Bioanalyzer 2100 System, and three biological replicates per group were sequenced by Novogene Co., Ltd. (Beijing, China). The DEGs between groups were screened using FC > 2 and *P*_adj_ < 0.05 as thresholds and functionally annotated by GO analysis.

### 2.8. UPLC-MS/MS Analysis

#### 2.8.1. UPLC/MS Conditions

UPLC was performed on the UPLC-Q-Exactive MS system (Thermo Fisher Scientific, California, USA) using a Halo-C18 column (2.1 mm × 100 mm, 2.7 *μ*m, AMT). The mobile phase was a mixture of distilled water (*A*) and acetonitrile (*B*) containing 0.05% formic acid. The sequence was as follows: 0–3 min, 0–2% *B*; 3–9 min, 2–40% *B*; 9–18 min, 40–98% *B*; and 18–21 min, 98% *B*. The sample loading amount was 2 *μ*l, the column temperature was set at 45°C, and the flow rate was set at 0.30 ml/min. MS analysis was performed in both positive and negative ionization modes equipped with a heated electrospray ionization source and Xcalibur 3.0 software. Optimal analysis conditions of the MS were set as follows: sheath gas 45 arb and auxiliary gas 10 arb; capillary voltage in positive and negative ion modes at 3500 V and 3000 V; capillary temperature 350°C; mass range 80–1000 m/z with a resolution of 70000; and S-lens RF level 55.

#### 2.8.2. Data Processing

To ensure data stability and reproducibility, 10 *μ*l of each serum sample was evenly mixed to make quality control (QC) samples. The acquired mass spectrometry data (.raw) were first converted to the mzXML format, and the peak identification, peak alignment, retention time, and peak area were extracted using R software. Peaks with missing values greater than 50% were filtered. HMDB (https://www.hmdb.ca/), METLIN (https://www.metlin.scripps.edu/), and KEGG (https://www.genome.jp/kegg/) databases and Xcalibur 3.0 software were used to identify metabolites. The absolute value of the mass error was less than 10 ppm. Principal component analysis (PCA) and orthogonal partial least squares discriminant analysis (OPLS-DA) were used to find METs. The MetaboAnalyst 5.0 (https://www.metaboanalyst.ca/) online platform was used for metabolic pathway analysis.

### 2.9. Integrated Transcriptomics and Metabolomics

The MetScape module of Cytoscape software, based on the KEGG database, was used to build the network of DEGs and potential metabolites. The target genes corresponding to the METs were searched in the HMDB database. The protein-protein interaction (PPI) network of DEGs and the gene targets of potential METs was constructed using the STRING database with a confidence score of 0.7.

### 2.10. Real-Time Quantitative PCR

Total aortic RNA was reverse transcribed to cDNA using the Evo M-MLV Mix Kit (Accurate Biology, Hu nan, China). RT-PCR was performed on the QuantStudio™5 system (Thermo Fisher, USA). The primer sequences are shown in Table 1.

### 2.11. Statistical Analysis

All data were analyzed using SPSS 22.0 software (SPSS Inc., Chicago, IL, USA) and visualized using GraphPad Prism 8.0 software (GraphPad Software, San Diego, CA, United States). Multiple groups were compared using one-way analysis of variance and Tukey's post hoc test and *P* < 0.05 was considered statistically significant.

## 3. Results

### 3.1. RS Administration Decreased BP in Prehypertensive Rats by Improving Endothelial Function

**Figure 1:**
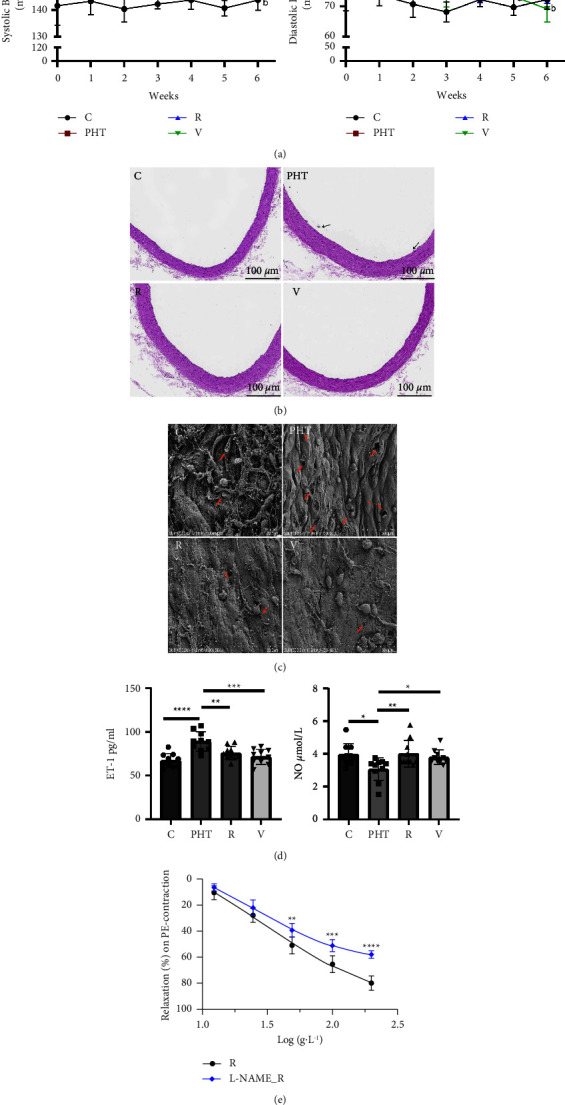
RS ameliorated endothelial dysfunction and reduced BP in prehypertensive rats. (a) BP in the indicated groups after 6 weeks of intragastric administration of RS. Data are expressed as mean ± SD (*n* = 10). ^a^*P* < 0.05, ^b^*P* < 0.01 vs. the PHT group. (b) Representative images of HE-stained thoracic aorta sections. (c) SEM images showing structural changes in the thoracic aorta. (d) Serum ET-1 and NO levels in the indicated groups. The data are expressed as mean ± SD (*n* = 10/group). ^*∗*^*P* < 0.05, ^*∗∗*^*P* < 0.01, ^*∗∗∗*^*P* < 0.001, ^*∗∗∗∗*^*P* < 0.0001 vs. the PHT group. (e) Relaxation rate of thoracic aorta rings after PE-precontraction or after preincubation with L-NAME. Results are mean ± SD (*n* = 6). ^*∗∗*^*P* < 0.01, ^*∗∗∗*^*P* < 0.001, ^*∗∗∗∗*^*P* < 0.0001 vs. RS alone. (C, control group; PHT, model group; R, RS-treated group; V, valsartan-treated group).

The initial BP of the prehypertensive rats was 160/85 mmHg compared to 140/70 mmHg in normal controls, indicating a successful model. The intervention with RS significantly decreased both SBP and DBP compared to those in the untreated PHT rats. The therapeutic effects of valsartan were manifested earlier and the SBP showed an obvious decrease after four weeks (^b^*P* < 0.01) (Figure 1(a)). Given the crucial role of the vascular endothelium in prehypertension, we next evaluated the effect of RS on the aortic endothelium. As shown in Figure 1(b), the endothelium of the thoracic aorta of PHT animals was partially disintegrated (marked by black arrows) and showed significant degeneration of the endothelial cells (ECs) and vascular wall thickening. RS intervention decreased the exfoliation of the ECs and restored the structural integrity of the vascular endothelium. SEM examination of the aortas (Figure 1(c)) further revealed a well-ordered, rope-shaped vascular endothelium in the control group, with interconnected ECs and minimal damage (marked by red arrows). In contrast, ECs in the PHT group showed extensive shedding and loss of intercellular junctions, resulting in cavities. However, the intervention with RS significantly reduced endothelial abscission. Taken together, RS alleviated the prehypertensive symptoms in rats by restoring the aortic vascular endothelium.

### 3.2. RS-Induced Vasodilation by Modulating ET-1 and NO Levels

Endothelins are potent vasoconstrictors, whereas endothelium-derived NO is the most powerful vasodilator. Lowered NO bioavailability and increased production of vasoconstrictor substances eventually lead to vascular ED [28]. The PHT rats had significantly higher serum ET-1 levels compared to controls, which decreased after treatment with RS and valsartan. On the contrary, NO levels were markedly reduced in PHT animals and restored by RS and valsartan interventions. Furthermore, RS elevated serum NO more effectively compared to valsartan (Figure 1(d)). This strongly indicated that RS can reverse vascular ED in prehypertensive rats by inducing vasodilation. To validate this hypothesis, we tested the effect of RS on the thoracic aorta in vitro using the vascular ring tension method. A cumulative gradient of RS had no significant effect on the tension of the rat aortic ring at rest. However, RS significantly relaxed the aorta precontracted with PE in a dose-dependent manner, and the maximum relaxation rate was approximately 80%. L-NAME is a nonselective inhibitor of endothelial nitric oxide synthase (eNOS), which can block the synthesis of NO and lead to vasoconstriction. As shown in Figure 1(e), pretreatment with L-NAME (100 mM) significantly inhibited the relaxation curve of the vascular rings in the presence of RS. This suggests that hypertension-related ED may involve an altered basal release of NO and that the antihypertensive effects of RS rely on NO-mediated vasodilation.

### 3.3. Transcriptomic Alterations with RS Treatment

**Figure 2:**
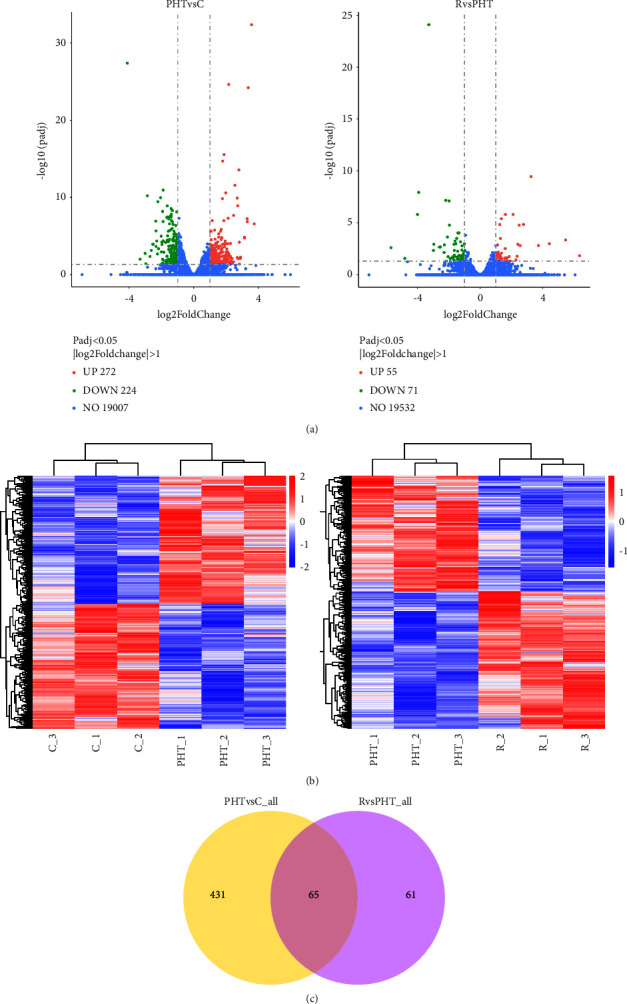
Gene expression profiling of prehypertensive rats. (a) Volcano plot of DEGs in the indicated groups. The upregulated, downregulated, and unaltered genes are shown as red, green, and blue dots, respectively. (b) Hierarchical clustering of DEGs. (c) Venn diagram showing intersecting DEGs after RS treatment.

**Figure 3:**
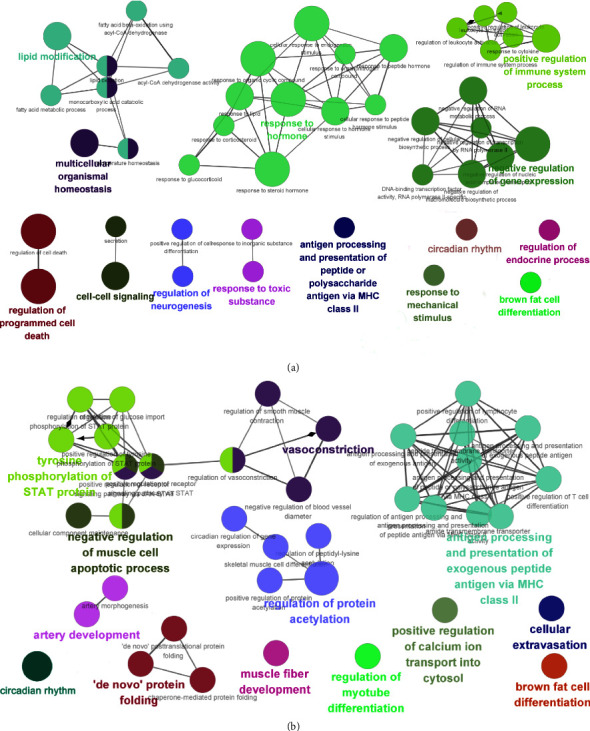
GO enrichment analysis of DEGs. (a) Enriched biological processes of DEGs between the model group and the control group. (b) Enriched biological processes of DEGs between the RS-treated group and the model group.

To further dissect the molecular mechanisms underlying the antihypertensive effects of RS, we compared the transcriptomes of the thoracic aorta of different groups. Using *P*_adj_ < 0.05 and FC > 2, we identified 496 DEGs between the PHT and the control group (PHT vs. C), of which 272 genes were upregulated and 224 genes were downregulated in the former. RS significantly upregulated 55 genes and downregulated 71 genes in the PHT group (Figure 2(a)). Furthermore, the gene expression profile of the RS-treated animals was closer to that of the controls than that of the untreated PHT animals (Figure 2(b)), indicating that RS also reversed the molecular changes associated with prehypertension. A Venn diagram of the DEGs between the two datasets (Figure 2(c)) revealed 65 DEGs in response to RS, of which 34 were upregulated and 31 were downregulated (Table 2). Gene Ontology (GO) analysis further indicated that the 496 DEGs were enriched for significant pathways, including “response to the hormone,” “antigen processing and presentation of peptide,” “response to a toxic substance,” “circadian rhythm,” “response to mechanical stimulus,” etc. (Figure 3(a)). Compared to the PHT group, the significantly enriched GO terms in the *R* group included “vasoconstriction,” “regulation of protein acetylation,” “antigen processing and presentation of peptide,” “circadian rhythm,” and “negative regulation of muscle cell apoptotic process” (Figure 3(b)). Taken together, circadian rhythm, antigen processing, and presentation of the peptide are crucial determinants of the antihypertensive effects of RS.

### 3.4. RS Affects Multiple Metabolic Pathways in Hypertensive Rats

**Figure 4:**
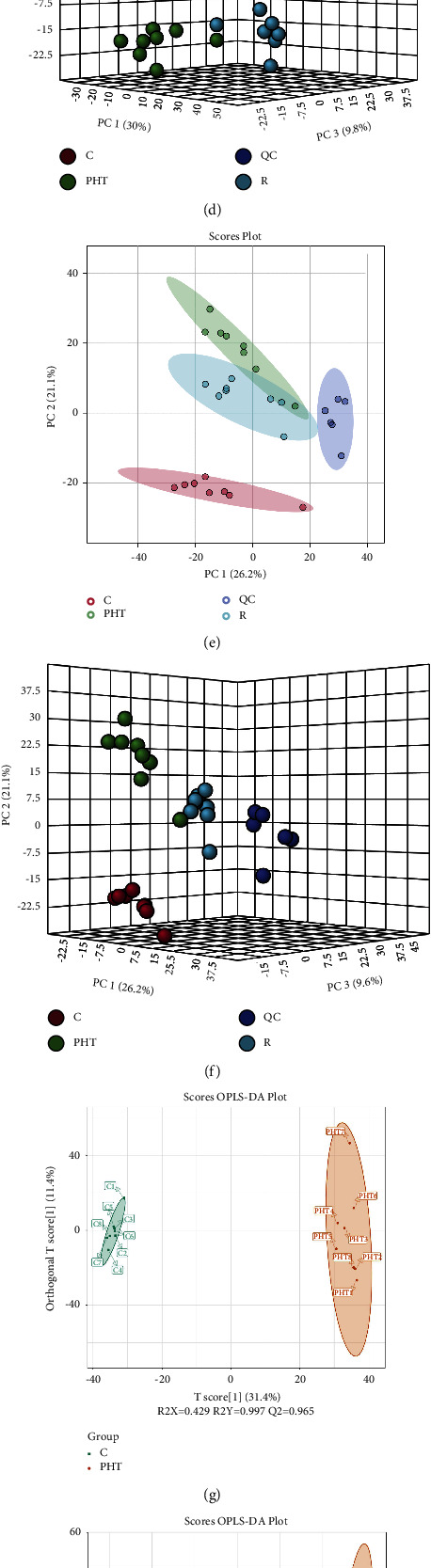
Detection of serum metabolites. TIC of serum of the indicated groups in positive (a) and negative (b) ion modes. 2D and 3D PCA plots based on data from the different groups obtained in the positive (c, d) and negative (e, f) ion modes. OPLS-DA score plots based on data from the different groups obtained in the positive (g, h) and negative (i, j) ion modes. C represents the control group, PHT represents the model group, R represents RS-treated group, and QC represents the quality control group.

The metabolic changes induced by RS were determined by comparing the serum metabolite profiles of the different groups. Total ion chromatograms (TIC) of sera from the control, PHT, and RS-treated groups are shown in Figures 4(a) and 4(b), which indicate a clear separation of metabolites along with differences in some peak intensities. PCA further reiterated the differences between the groups through data dimensionality reduction (Figures 4(c)–4(f)). Furthermore, analysis of the QC samples showed that the detection method had good reproducibility and stability and that our data were reliable. Good separation was achieved between the control and PHT groups, indicating that prehypertension altered the metabolic profile, resulting in significant differences in the content of endogenous metabolites. On the other hand, the metabolites of the *R* group were closer to those of the control group, suggesting that the RS intervention improved metabolism. OPLS-DA was performed to identify metabolites that can discriminate between samples (Figures 4(g)–4(j)) on the basis of variable importance in projection (VIP). METs were screened using VIP > 1, *P* value < 0.05, and FC ≥ 2 or ≤0.5 as the thresholds. As shown in Table 3, there were 52 potential METs after RS intervention, which were functionally annotated using the MetaboAnalyst 5.0 platform. The METs were enriched in 17 metabolic pathways (impact > 0.01), including linoleic acid metabolism, selenocompound metabolism, D-glutamine, and D-glutamate metabolism, arginine biosynthesis, histidine metabolism, arginine and proline metabolism, purine metabolism, pentose and glucuronate interconversions, sphingolipid metabolism, and so on (Table 4). Taken together, RS exerts its antihypertensive effects by targeting multiple pathways.

### 3.5. Integrated Transcriptomics and Metabolomics

**Figure 5:**
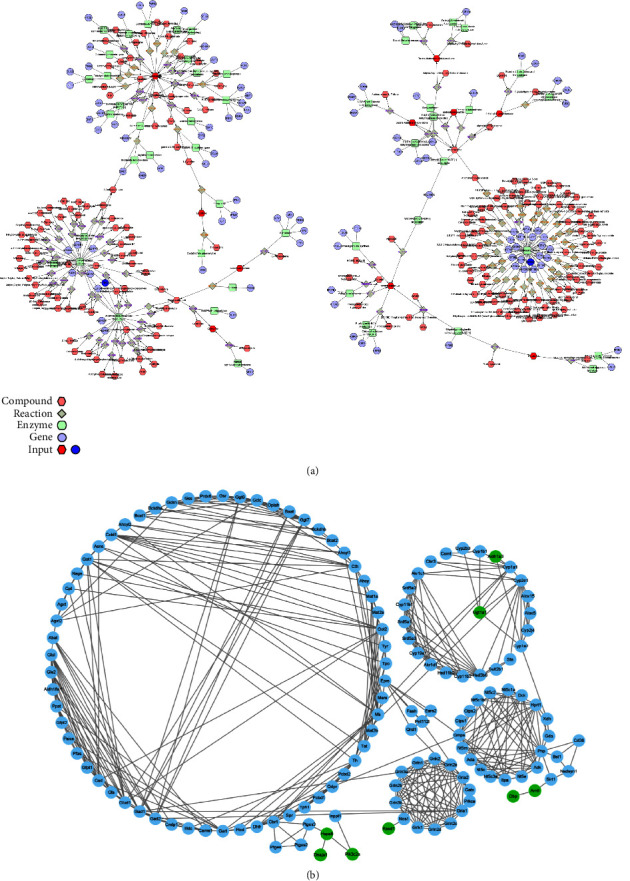
Integrated transcriptomics and metabolomics. (a) Network analysis map of DEGs and METs. (b) PPI network diagram based on STRING. Green dots represent DEGs and blue dots represent gene targets of potential METs.

The network of DEGs and METs was then constructed to explore their causal relationship in response to RS (Figure 5(a)). Ugt1a1 interacted with androstanedione and androsterone sulfate through androgen and estrogen biosynthesis and metabolism pathways under the action of glucuronosyltransferase, and was associated with 7alpha-hydroxycholest-4-en-3-one through bile acid biosynthesis. Additionally, prostaglandin E2 and lipoxin A4 were formed from arachidonate by arachidonic acid (AA) metabolism with nicotinamide adenine dinucleotide (NAD) (+) or nicotinamide adenine dinucleotide phosphate (NADP) (+) as acceptors. Aldh1a3 interacted with 2-phenylacetamide and carnosine through tyrosine metabolism and histidine metabolism pathways, respectively, and interacted with L-glutamate and L-cystine through the urea cycle and metabolism. Due to the limited nature of MetScape, some metabolites and genes were not recognized. PPI network analysis using the STRING database revealed 448 nodes and 136 interacting pairs, with an average node degree of 5.83 and an average local clustering coefficient of 0.46. As shown in Figure 5(b), eight DEGs, including Pik3c2a, Hspa8, Dnaja1, Ugt1a1, Aldh1a3, Dbp, Rasd1, and Arntl, were associated with potential metabolites.

### 3.6. Verification of the Key Target Genes of RS in Prehypertensive Rats

**Figure 6:**
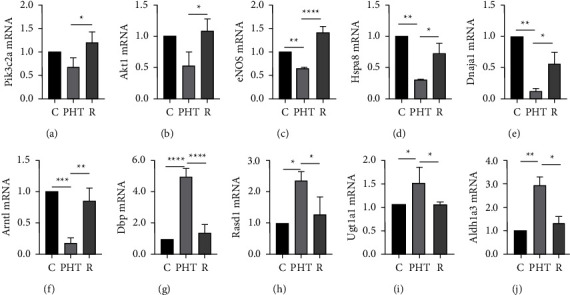
qRT-PCR for target genes using divergent primers. The mRNA levels of Pik3c2a (a), Akt1 (b), eNOS (c), Hspa8 (d), Dnaja1 (e), Arntl (f), Dbp (g), Rasd1 (h), Ugt1a1 (i), and Aldh1a3 (j) were detected. ^*∗*^*P* < 0.05, ^*∗∗*^*P* < 0.01, ^*∗∗∗*^*P* < 0.001, and ^*∗∗∗∗*^*P* < 0.0001 vs. the PHT group.

The target genes of RS identified above were validated in the thoracic aorta by qRT-PCR. As shown in Figure 6, Pik3c2a, Akt1, eNOS, Hspa8, Dnajal, and Arntl were downregulated, and Dbp, Rasd1, Ugt1a1, and Aldh1a3 were upregulated in the PHT group compared to the controls. Pik3c2a and Akt1 did not show a significant difference, but they showed a downward trend in the PHT group. RS treatment increased the transcript levels of Pik3c2a, Akt1, eNOS, Hspa8, Dnajal, and Arntl and decreased those of Dbp, Rasd1, Ugtlal, and Aldh1a3. Interestingly, Akt1 showed a trend similar to that of Pik3c2a, while eNOS mRNA was significantly downregulated in the PHT group and increased with RS treatment. These findings further support our hypothesis that RS alleviates hypertension through NO-mediated vasodilation.

## 4. Discussion

Prehypertensive patients are at a higher risk of developing hypertension and CVD compared to those with normal BP [29], but are often overlooked. ED is the pathological basis of hypertension and prehypertension, which was also established in the prehypertensive rat model in terms of elevated ET-1 and lower NO levels in sera. The RS intervention reversed these changes and also repaired the damaged aortic endothelium. The vascular ring assay showed that RS reduced BP by improving relaxation of the vascular endothelium. Furthermore, the vasodilatory effect of RS on the thoracic aorta of a prehypertensive rat was confirmed by the increased vasoconstriction seen after preincubating the blood vessels with the eNOS inhibitor L-NAME.

Early metabolomics studies have shown that the serum metabolite profile of individuals with borderline hypertension is distinct from that of hypertensive patients [30]. This strongly indicated that the prehypertensive stage is associated with changes in specific metabolites that can potentially be used for early diagnosis. We identified 52 METs in the sera of RS-treated hypertensive rats, most of which have been reported previously to be related to prehypertension or hypertension. To compensate for the limitations of untargeted metabolomics, we integrated the transcriptomes and metabolomes to construct a network and identify the key targets of drug intervention. Eight putative RS targets were related to pathways involved in BP regulation and may be promising biomarkers for early diagnosis and risk assessment.

RS may exert its effects through the phosphatidylinositol signaling pathway, which plays an important role in BP regulation. Pik3c2a, a member of the phosphoinositide 3-kinases (PI3K) family, phosphorylates the 3-hydroxyl of the phosphatidylinositol (PI) ring, producing a second messenger that relays signals via multiple pathways. In addition, Pik3c2a is critical for EC junctions [31] and survival [32, 33], and its absence can alter platelet structure and viscosity through shear stress [34]. Laminar shear stress increases the interaction between Sirt1 and eNOS, as well as eNOS deacetylation, to enhance NO production and reverse ED [35]. Phosphoinositide-dependentkinase-1 (PDK1) interacts with phosphatidylserine (PS) and phosphatidylinositol-3,4,5-trisphosphate (PIP_3_) through different kinase domains and activates Akt [36]. Akt1 is the main subtype of Akt, which is expressed in ECs and preferentially phosphorylates eNOS and promotes NO release in vitro [37]. The levels of PS, Pik3c2a, Akt1, and eNOS were significantly increased after RS intervention, indicating that it improves endothelial function and reduces BP by regulating phosphoinositol signaling.

Elevated ET-1 during hypertension increases vascular superoxide levels, which further aggravates ED. The main sources for oxidative excess in the vasculature are NADPH oxidase, xanthine oxidase (XOD), and uncoupled eNOS [28, 38]. XOD directly oxidizes xanthine to produce superoxide anion and uric acid (UA), which is the final product of purine degradation and is depleted in the absence of purine-nucleoside phosphorylase (PNP). Enhanced levels of superoxide anions also decrease NO bioavailability and further promote ED. Studies show that UA independently increases the risk of prehypertension [39]. Therefore, lowering plasma UA levels may prevent prehypertension [40]. Xanthine and adenosine levels decreased, and those of xanthosine increased significantly after RS treatment. Therefore, the therapeutic effect of RS can also be attributed to the regulation of the purine metabolism pathway.

Most physiological functions in humans, including BP regulation, follow circadian rhythms. Disturbance of endogenous circadian rhythms increases the risk of hypertension [41]. The transcription factors BMAL1 and CLOCK form a heterodimer that drives the transcription of circadian genes (Per and Cry), tissue-specific genes like Edn1, and clock-controlled genes such as Dbp by binding to E-Box response elements. BMAL1, also known as aryl hydrocarbon receptor nuclear translocator-like (Arntl) protein, is downregulated in hypertensive women [42]. Studies show that PPAR*γ* activator reduces BP by inducing the aortic expression of BMAL1 mRNA [43]. Compared to hypertensive rats, the expression levels of BMAL1 and CLOCK are higher in WKY rats [44]. Arntl levels increased after RS intervention, indicating that it may be critical for its antihypertensive effects. Circulating ET-1, the product of the Edn1 gene, also exhibits a circadian rhythm [45]. Through a negative feedback loop, Per and Cry inhibit their own transcription by forming a complex with the BMAL1-CLOCK heterodimer. In addition to the negative autoregulatory feedback loop of Per and Cry, the Dbp-mediated loop also amplifies the circadian oscillation. Sirt1 regulates the circadian rhythm by histone deacetylation [46] and increases the expression of endothelial NO [47], which is negatively correlated with Dbp [44]. RS significantly reduced the level of Dbp, which may reduce BP by upregulating eNOS. Rasd1, also known as Dexras1, regulates circadian rhythm in response to external signals and can also activate physiological NO signaling [48]. Furthermore, Rasd1 can inhibit the activity of cyclic adenosine monophosphate (cAMP) [49]. Thus, RS-mediated downregulation of Rasd1 may reverse the inhibition of cAMP and relax the blood vessels [50].

Linoleic acid (LA), a precursor of AA biosynthesis, is an essential fatty acid that maintains physiological levels of prostaglandins and thromboxanes and regulates vascular tone [51]. LA is metabolized by various enzymes, including cytochrome P450 (CYP), lipoxygenase (LO), and cyclooxygenase (COX), of which CYP1A2 has a strong catalytic activity. Previous studies have shown that hypertensive rats have lower levels of LA and a higher level of AA compared to normotensive controls due to inhibition of COX activity [52]. Therefore, restoring the levels of both compounds can prevent hypertension [53] through improved NO bioavailability and amelioration of ED [54]. Thus, an RS-mediated increase in NO levels can be due to the increase in LA, which may be one of the active substances regulating BP.

Eicosanoid metabolites are produced by AA metabolism in blood vessels, and their levels in CVD are the major determinants of ED [51]. AA is also metabolized through the CYP, LO, and COX pathways. The products of the CYP-dependent metabolism of AA are associated with increased renal vascular resistance in prehypertensive SHR [55]. The COX-mediated synthesis of prostaglandin E2 (PGE2) is diminished in prehypertensive rats [56]. The effect of PGE2 on blood vessels depends on its receptor [57]. For instance, after binding to the receptors EP2 and EP4, PGE2 promotes cAMP production and induces vascular smooth muscle relaxation [50]. RS probably exerts its antihypertensive effect by increasing PGE2 levels. Prostaglandin *E* synthase 3 (PTGES3) catalyzes the oxidoreduction of prostaglandin H2 (PGH2) to PGE2, which functions as a cochaperone with heat shock protein 90. Dnaja1 encodes Hsp40 proteins, which act as cochaperones for Hspa8. Overexpression of Hspa8 inhibited EC apoptosis and promoted angiogenesis and vascular remodeling following vascular injury [58]. Genetic variations in Hspa8 correlate inversely with the risk of hypertension [59]. Furthermore, Hspa8 is also upregulated in patients with arterial hypertension, which may be a protective response to EC injury [60]. The expression of Hspa8 and Dnaja1 increased in the RS-treated group, which probably promoted the transformation of PGH2 to PGE2 via PTGES3, eventually lowering BP.

We also observed an upregulation of 12(R)-HETE and a downregulation of lipoxin A4(LXA4), which are AA metabolites produced via the LO pathway in vascular ECs. A previous study showed that 12(R)-HETE acts as an agonist of the vasodilator in preconstricted mouse artery [61]. LXA4 has anti-inflammatory, vasodilatory, and antioxidant effects [62], and low LXA4 levels may be an indicator of the development of hypertension [63]. On the contrary, increased LXA4 in the plasma of preeclampsia women plays a central role in disease development [64].

UDP glucuronosyltransferase family 1 member A1 (Ugt1a1) is the only enzyme that can metabolize bilirubin and control serum bilirubin levels. Bilirubin is the end product of heme catabolism and a risk factor for CVD [65]. Genetic variations in Ugt1a1 are associated with elevated bilirubin and an increased risk of hypertension in individuals with African ancestry [66]. Mice treated with anti-Ugt1a1 antibodies show a reduction in BP, which can be attributed to lower oxidative stress and increased NO levels [67]. Ugt1a1 catalyzes glucuronidation at the 3-OH of dihydrocaffeic acid (DHCA), which scavenges reactive oxygen species in ECs and increases the level of eNOS to protect the ECs from oxidative damage [68]. We found that RS downregulated Ugt1a1 and increased DHCA levels, indicating that RS can exert an antihypertensive function by increasing the level of eNOS.

Aldehyde dehydrogenase family 1 member 3 (Aldh1a3) regulates proliferation in the neointima and the medial layer of vascular smooth muscle [69]. Inhibition of excessive Aldh1a3 activity can alleviate intimal hyperplasia. Additionally, a variation in the Aldh1a3 gene has been linked to the hydrochlorothiazide response in hypertensive individuals [70], and the increased Aldh1a3 led to intimal hyperplasia and lumen stenosis in a rat model of carotid artery injury [71]. The expression of Aldh1a3 decreased after administration of RS, indicating that it may lower BP by reducing Aldh1a3 expression.

## 5. Conclusions

**Figure 7:**
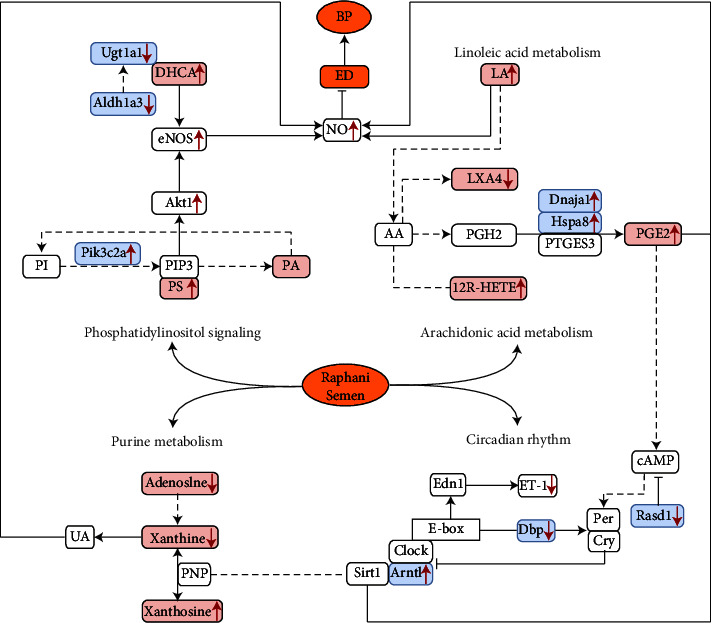
Framework diagram of key links and mechanisms of RS intervention in prehypertensive rats.

RS can effectively reduce the BP of prehypertensive rats and improve vascular ED by regulating purine, linoleic acid, AA metabolism, circadian rhythm, and the phosphatidylinositol signaling pathway. Pik3c2a, Hspa8, Dnaja1, Arntl, Ugt1a1, Dbp, Rasd1, and Aldh1a3 may be the key genes targeted by RS intervention in prehypertension (Figure 7). More research is needed to validate the clinical potential of our findings.

## Figures and Tables

**Table 1:** Rat primers sequence used for qRT-PCR analysis.

Gene name	Primer sequence (5′ to 3′)	
*β*-Actin	Forward	TGTCACCAACTGGGACGATA
*β*-Actin	Reverse	GGGGTGTTGAAGGTCTCAAA
Pik3c2a	Forward	CCAGAATGGCTTCAGTCCAAGGATG
Pik3c2a	Reverse	AATGGTGTGGCAGGTGTCAAAGG
eNOS	Forward	GCCACCTGATCCTAACTTGCCTTG
eNOS	Reverse	TCGTGTAATCGGTCTTGCCAGAATC
Akt1	Forward	CACAGGTCGCTACTATGCCATGAAG
Akt1	Reverse	GCAGGACACGGTTCTCAGTAAGC
Arntl	Forward	CGTGCTCAGGATGGCTGTTCAG
Arntl	Reverse	AGGTGCAGTGTCCGAGGAAGATAG
Dbp	Forward	CACCGCTTCTCAGAGGAGGAATTG
Dbp	Reverse	CCTCTTGGCTGCTTCATTGTTCTTG
Rasd1	Forward	GAAGATGTGCCCAAGCGACTCTG
Rasd1	Reverse	CACTTTGGATGAGCCGAGGATGAC
Dnaja1	Forward	AATGTCGTGCATCAGCTCTCAGTG
Dnaja1	Reverse	CTTTCTTACCACCTCGGCCTTCAC
Hspa8	Forward	TCAGGATTTGCTGCTCTTGGATGTC
Hspa8	Reverse	TGCTTGGTGGGAATGGTGGTATTG
Aldh1a3	Forward	TCAACAATGACTGGCACGAACCC
Aldh1a3	Reverse	GCTTATCGCCTTCTTCCACCTCAC
Ugt1a1	Forward	CTCGGGCGTTCATCACACACTC
Ugt1a1	Reverse	TCACCAAACAAGGGCATCATCACC

**Table 2:** Differentially expressed genes in response to RS.

Gene ID	Gene name	Gene description	PHT vs C			*R* vs PHT		
			Log_2_FC	*P*_adj_	Trend	Log_2_FC	*P*_adj_	Trend
ENSRNOG00000021027	Dbp	D-box binding PAR bZIP transcription factor	3.567303898	4.30*E* − 33	Up	−3.264046382	7.90*E* − 25	Down
ENSRNOG00000046202	Metrnl	Meteorin-like, glial cell differentiation regulator	1.969308729	2.56*E* − 11	Up	−1.34813636	9.33*E* − 05	Down
ENSRNOG00000027722	H1fx	H1 histone family, member *X*	1.851322912	1.01*E* − 07	Up	−1.394696117	0.031399605	Down
ENSRNOG00000008862	Abcg4	ATP binding cassette subfamily G member 4	1.655409762	2.47*E* − 06	Up	−1.956800064	1.68*E* − 05	Down
ENSRNOG00000021183	LOC102552640	REST corepressor 2-like	1.716720712	1.59*E* − 05	Up	−1.588589807	0.000560329	Down
ENSRNOG00000045797	Lep	Leptin	1.947370701	4.43*E* − 05	Up	−2.081133473	0.000226691	Down
ENSRNOG00000012172	Spi1	Spi-1proto-oncogene	1.321833554	0.000127507	Up	−1.03255045	0.026766336	Down
ENSRNOG00000020030	Crlf1	Cytokine receptor-like factor 1	1.004675242	0.000150727	Up	−1.489669973	0.00030577	Down
ENSRNOG00000021041	Fam171a2	Family with sequence similarity 171, member A2	1.107709992	0.000154887	Up	−1.164578955	0.004227803	Down
novel.1017	—	PF00249: Myb-likeDNA-binding domain	1.763815781	0.000331296	Up	−1.607636495	0.018048572	Down
ENSRNOG00000058006	Sncg	Synuclein, gamma	1.260680962	0.000372941	Up	−1.667606306	0.001152749	Down
ENSRNOG00000024923	Nnat	Neuronatin	1.025714286	0.000425846	Up	−2.187827367	6.74*E* − 08	Down
ENSRNOG00000017463	Bloc1s3	Biogenesis of lysosomal organelles complex-1, subunit 3	1.734624953	0.000454039	Up	−1.014924909	0.036667207	Down
ENSRNOG00000008536	Actc1	Actin, alpha, cardiac muscle 1	2.041299184	0.000480238	Up	−3.909775367	1.13*E* − 08	Down
ENSRNOG00000048961	Bhlhe41	Basic helix-loop-helix family, member e41	1.714013346	0.000532216	Up	−1.459316165	0.027634003	Down
ENSRNOG00000010575	Dapp1	Dual adaptor of phosphotyrosine and 3-phosphoinositides 1	1.140266497	0.001212619	Up	−1.100294229	0.033236941	Down
ENSRNOG00000018735	Cd74	CD74 molecule	1.112172854	0.001238105	Up	−1.05684107	0.045825102	Down
ENSRNOG00000032844	RT1-Da	RT1 class II, locus Da	1.261046152	0.001903907	Up	−1.213526605	0.020265998	Down
ENSRNOG00000018740	Ugt1a1	UDP glucuronosyltransferase family 1 member A1	1.058435351	0.00383177	Up	−1.155931747	0.001591236	Down
ENSRNOG00000032639	Foxo6	Forkhead box O6	1.849242891	0.003855283	Up	−2.270257913	0.001325659	Down
ENSRNOG00000011478	Ackr4	Atypical chemokine receptor 4	1.107026643	0.006340248	Up	−1.53929622	0.001046107	Down
ENSRNOG00000019330	Procr	Protein C receptor	1.022734389	0.006435929	Up	−1.086629096	0.015518083	Down
ENSRNOG00000003144	Gprc5c	G protein-coupled receptor, class C, group 5, member C	1.132504804	0.006805995	Up	−1.429058886	0.005581253	Down
ENSRNOG00000000451	RT1-Ba	RT1 class II, locus Ba	2.226046253	0.007035112	Up	−2.588878749	0.002146712	Down
ENSRNOG00000046560	AC109096.1	—	1.075351448	0.009966035	Up	−1.011598656	0.036021456	Down
ENSRNOG00000055564	RGD1564664	Similar to LOC387763 protein	1.294671413	0.015607613	Up	−1.711461574	0.032923765	Down
ENSRNOG00000003348	Rasd1	Ras-related dexamethasone induced 1	1.16515603	0.016140158	Up	−1.364279536	0.04658816	Down
ENSRNOG00000061998	AABR07044421.1	—	1.681819659	0.01966356	Up	−3.988901029	1.55*E* − 06	Down
ENSRNOG00000020129	Cdh3	Cadherin 3	1.574152395	0.025609303	Up	−2.943616786	0.001152749	Down
ENSRNOG00000052070	Aldh1a3	Aldehyde dehydrogenase 1 family, member A3	1.049569581	0.033518518	Up	−1.348015788	0.013982309	Down
ENSRNOG00000017786	Acta1	Actin, alpha 1, skeletal muscle	1.400082213	0.036004573	Up	−2.147189191	0.049136448	Down
ENSRNOG00000029980	Zbtb16	Zinc finger and BTB domain containing 16	−4.106676387	4.01*E* − 28	Down	3.244177922	3.38*E* − 10	Up
ENSRNOG00000003669	Myocd	Myocardin	−2.866059593	6.36*E* − 11	Down	2.101360276	1.55*E* − 06	Up
ENSRNOG00000007029	Dnaja1	DnaJ heat shock protein family (Hsp40) member A1	−2.220517729	3.65*E* − 10	Down	1.610706495	1.55*E* − 06	Up
ENSRNOG00000026643	Chordc1	Cysteine and histidine rich domain containing 1	−1.60790284	4.43*E* − 05	Down	1.359311638	4.13*E* − 06	Up
ENSRNOG00000003666	Jchain	Immunoglobulin joining chain	−1.932062043	0.03948545	Down	2.75879606	1.40*E* − 05	Up
ENSRNOG00000017864	Bdp1	B double prime 1, subunit of RNA polymerase III transcription initiation factor IIIB	−1.407811425	2.14*E* − 08	Down	1.305094452	0.000312668	Up
ENSRNOG00000014248	Erbb4	Erb-b2 receptor tyrosine kinase 4	−2.289119562	4.43*E* − 05	Down	2.399656764	0.001152749	Up
ENSRNOG00000014509	Sacs	Sacsin molecular chaperone	−1.547973674	5.71*E* − 08	Down	1.116175993	0.001325659	Up
novel.811	—	—	−2.332034287	0.000225106	Down	2.543235677	0.001532242	Up
ENSRNOG00000011358	Hipk3	Homeodomain-interacting protein kinase 3	−1.03711145	0.000286261	Down	1.157986776	0.006627494	Up
ENSRNOG00000021525	Nbeal1	Neurobeachin-like 1	−1.132308326	0.000246838	Down	1.136606099	0.007849647	Up
ENSRNOG00000013587	Fam135a	Family with sequence similarity 135, member A	−1.641150683	1.30*E* − 09	Down	1.371986259	0.008457281	Up
ENSRNOG00000007706	Prkaa2	Protein kinase AMP-activated catalytic subunit alpha 2	−1.344153744	1.07*E* − 06	Down	1.197473792	0.010909631	Up
ENSRNOG00000061862	Zbtb10	Zinc finger and BTB domain containing 10	−1.496108262	1.89*E* − 06	Down	1.151806982	0.015116305	Up
ENSRNOG00000014448	Arntl	Aryl hydrocarbon receptor nuclear translocator-like	−1.57376632	2.47*E* − 06	Down	1.304448787	0.017388842	Up
ENSRNOG00000056135	Tsc22d3	TSC22 domain family, member 3	−1.122847202	4.32*E* − 07	Down	1.111465323	0.017814725	Up
ENSRNOG00000005957	Slc4a7	Solute carrier family 4 member 7	−1.091006955	0.013775843	Down	1.352856498	0.023295922	Up
ENSRNOG00000034269	Ago3	Argonaute RISC catalytic component 3	−1.280325929	0.005014064	Down	1.291240162	0.025121895	Up
ENSRNOG00000010065	Dgkh	Diacylglycerol kinase, eta	−1.955551832	0.000127205	Down	1.637806704	0.025121895	Up
ENSRNOG00000056716	Zbtb20	Zinc finger and BTB domain containing 20	−1.975779905	6.49*E* − 05	Down	1.636603951	0.026446922	Up
ENSRNOG00000003742	Cdkl5	Cyclin-dependentkinase-like 5	−1.88256747	1.15*E* − 11	Down	1.371912691	0.027923496	Up
ENSRNOG00000020479	Pik3c2a	Phosphatidylinositol-4-phosphate 3-kinase, catalytic subunit type 2 alpha	−1.114519009	5.97*E* − 06	Down	1.008209449	0.031399605	Up
ENSRNOG00000034066	Hspa8	Heat shock protein family A (Hsp70) member 8	−1.07279022	0.003973704	Down	1.003978216	0.032923765	Up
ENSRNOG00000011619	Myo9a	Myosin IXA	−1.08677208	0.000331296	Down	1.010547893	0.033236941	Up
ENSRNOG00000013884	Psd3	Pleckstrin and Sec7 domain containing 3	−1.386129469	0.004016511	Down	1.325924895	0.037676589	Up
novel.55	—	—	−1.234762995	5.91*E* − 06	Down	1.037997999	0.040884285	Up
ENSRNOG00000042519	Peak1	Pseudopodium-enriched atypical kinase-1	−1.121812695	0.005693797	Down	1.274772342	0.043162444	Up
ENSRNOG00000018804	Ripor2	RHO family-interacting cell polarization regulator 2	−1.624879022	9.28*E* − 06	Down	1.141179168	0.045191834	Up
ENSRNOG00000010640	Agtr1b	Angiotensin II receptor, type 1b	−2.329759002	2.24*E* − 06	Down	1.702703791	0.04658816	Up
ENSRNOG00000014037	Dcun1d3	Defective in cullin neddylation 1 domain containing 3	−1.063960359	0.007204179	Down	1.083854353	0.046969316	Up
ENSRNOG00000054515	Fgd6	FYVE, RhoGEF, and PH domain containing 6	−1.180943279	0.013459939	Down	1.413119049	0.048490736	Up
ENSRNOG00000042679	AABR07006860.1	Ligand-dependent nuclear receptor corepressor	−1.050601726	0.015762342	Down	1.209207063	0.048490736	Up
ENSRNOG00000045863	Naa16	*N* (alpha)-acetyltransferase 16, NatA auxiliary subunit	−1.006785492	0.030931715	Down	1.086757495	0.048490736	Up
ENSRNOG00000031092	Rock1	Rho-associatedcoiled-coil containing protein kinase-1	−1.410378076	0.001917703	Down	1.529559464	0.049136448	Up

**Table 3:** Identification of significant differential metabolites after the intervention of RS in model rats by UPLC-Q-exactive MS/MS.

No.	Ion mode	*tR*(min)	Formula	Measured mass *m*/*z*	MS2	Compound name	HMDB_ID	Error ppm	PHT vs. C	*R*vs. PHT
1	Pos	19.43	C_6_H_12_N_2_O_4_S_2_	241.0309 (M + H)	151.9835, 120.0116, 122.0272, 76.9286	L-Cystine	HMDB0000192	2.904	Down	Down
2	Pos	0.73	C_8_H_14_N_3_O_7_P	296.0657 (M + H)	104.1074, 236.9923, 86.097, 169.9858, 105.1107	5-Aminoimidazole ribonucleotide	HMDB0001235	5.742	Down	Up
3	Pos	1.50	C_8_H_16_O_2_	306.2636 (2M + NH_4_)	172.9535, 154.9421	Valproic acid	HMDB0001877	6.680	Up	Down
4	Pos	5.68	C_14_H_12_O_3_	251.0701 (M + Na)	56.9656, 205.0374, 228.0539	Resveratrol	HMDB0003747	6.891	Up	Down
5	Pos	0.78	C_5_H_9_NO_4_	192.0242 (M + 2Na − H)	151.035, 128.0191	L-Glutamic acid	HMDB0000148	3.854	Down	Up
6	Pos	4.83	C_10_H_12_N_4_O_6_	285.0828 (M + H)	153.0406, 133.0496	Xanthosine	HMDB0000299	2.455	Down	Up
7	Pos	0.84	C_7_H_14_N_2_O_3_	197.0896 (M + Na)	80.9485, 179.0903, 82.9456, 138.0524	N-Acetylornithine	HMDB0003357	2.892	Down	Up
8	Pos	14.74	C_20_H_32_O_5_	375.2138 (M + Na)	—	Prostaglandin E2	HMDB0001220	2.585	Up	Up
9	Pos	2.49	C_6_H_10_O_3_	175.0342 (M + 2Na − H)	74.9316, 115.9577, 133.9679, 128.951	4-Methyl-2-oxovaleric acid	HMDB0000695	3.085	Up	Down
10	Pos	1.76	C_6_H_6_N_2_O_2_	139.0422 (M + H)	84.9603, 77.0063, 81.0706, 95.0861, 102.9707	Nicotinamide-N-oxide	HMDB0002730	−7.994	Up	Down
11	Pos	1.24	C_4_H_9_NO_3_	120.0655 (M + H)	72.0815, 61.0405, 73.0848, 103.0547, 55.0552, 74.0607	L-Homoserine	HMDB0000719	4.164	Down	Up
12	Pos	12.88	C_18_H_37_NO_2_	300.2893 (M + H)	270.2787, 282.2787	3-Dehydrosphinganine	HMDB0001480	2.997	Down	Up
13	Pos	2.76	C_8_H_9_NO	199.0841 (M + CH_3_CN + Na)	154.0419, 156.0388, 155.0455	2-Phenylacetamide	HMDB0010715	3.114	Down	Up
14	Pos	11.03	C_13_H_20_O	239.1639 (M + H + HCOOH)	221.1535, 193.1586, 175.148	Alpha-ionone	HMDB0059883	3.261	Down	Up
15	Pos	17.50	C_42_H_78_NO_10_P	752.5192 (M + H − 2H_2_O)	184.0732, 146.9818	PS (18 : 1(9Z)/18 : 1(9Z))	HMDB0012390	5.023	Down	Up
16	Pos	14.01	C_20_H_30_O_3_	283.2053 (M + H − 2H_2_O)	265.1948, 223.1479	12-HEPE	HMDB0010202	3.107	Down	Up
17	Pos	15.17	C_20_H_34_O_3_	305.2471 (M + H − H_2_O)	119.0857, 93.0704, 105.0702, 133.1011, 147.1168, 175.1117	15S-Hydroxy-8Z, 11Z, 13E-eicosatrienoic acid	HMDB0005045	3.079	Down	Up
18	Pos	11.04	C_21_H_36_O_3_	405.2632 (M + Na + HCOOH)	363.1469, 351.2314	Pregnanetriol	HMDB0006070	3.825	Down	Up
19	Pos	16.56	C_27_H_44_O_2_	401.3411 (M + H)	383.3298, 97.0652, 177.1275, 384.3336, 95.0859, 81.0704	7-alpha-hydroxy-4-cholesten-3-one	HMDB0001993	1.993	Down	Down
20	Pos	15.76	C_22_H_34_O_2_	331.2627 (M + H)	105.0702, 119.0857, 91.0547, 131.0856, 81.0705	cis-7, 10, 13, 16, 19-Docosapentaenoic acid	HMDB0006528	3.019	Down	Up
21	Pos	8.86	C_42_H_83_O_8_P	810.6051 (M + CH_3_CN + Na)	114.0916, 209.1645, 322.2485, 228.1591	PA (20 : 0/i-19 : 0)	HMDB0115641	7.624	Down	Up
22	Pos	17.76	C_42_H_84_NO_8_P	806.5682 (M + 2Na − H)	184.0731, 86.0969, 185.0766	PE-NMe (18 : 0/18 : 0)	HMDB0112975	3.670	Down	Up
23	Pos	17.76	C_40_H_78_NO_8_P	754.5369 (M + Na)	184.0731, 59.0499, 86.097	PE-NMe (20 : 0/14 : 1(9Z))	HMDB0113280	0.967	Up	Down
24	Pos	13.69	C_20_H_32_O_6_	391.2087 (M + Na)	149.0235	Thromboxane B3	HMDB0005099	2.479	Up	Up
25	Pos	5.58	C_5_H_11_NO_2_Se	244.0108 (M + H + HCOOH)	156.0387, 202.044, 157.0422	Seleno-L-methionine	HMDB0003966	8.278	Up	Down
26	Pos	12.18	C_9_H_13_N_5_O_3_	222.0964 (M + H-H_2_O)	201.5828, 190.0749, 202.0844, 165.0489	7,8-Dihydro-L-biopterin	HMDB0000038	9.686	Up	Up
27	Pos	4.01	C_10_H_13_N_5_O_4_	268.1039 (M + H)	136.0619, 137.0459	Adenosine	HMDB0000050	2.611	Up	Down
28	Pos	11.32	C_26_H_43_NO_5_	467.3475 (M + NH_4_)	414.2999, 432.3105, 415.3034	Glycochenodeoxycholic acid	HMDB0000637	−0.961	Down	Up
29	Neg	10.27	C_9_H_14_N_4_O_3_	451.2052 (2M − H)	96.968	L-Carnosine	HMDB0000033	0.937	Up	Down
30	Neg	1.03	C_5_H_4_N_4_O_2_	187.0004 (M + Cl)	96.968, 57.974, 87.0071, 128.0338	Xanthine	HMDB0000292	9.893	Up	Down
31	Neg	1.47	C_6_H_12_O_5_	201.0162 (M − 2H + K)	164.8348, 162.8378	L-Fucose	HMDB0000174	4.527	Up	Down
32	Neg	10.49	C_24_H_40_O_5_	443.2571 (M + Cl)	121.028, 113.0228	Allocholic acid	HMDB0000505	1.466	Down	Up
33	Neg	9.20	C_9_H_10_O_4_	181.0495 (M − H)	96.9585, 61.9867, 153.9212	Dihydrocaffeic acid	HMDB0000423	0.137	Up	Up
34	Neg	10.11	C_12_H_22_O_11_	342.1155 (M−)	164.115, 163.1116, 165.1184	Kojibiose	HMDB0011742	2.046	Up	Down
35	Neg	16.93	C_24_H_40_O_3_	411.2675(M + Cl)	375.2904, 357.2799, 235.17, 376.2939	Lithocholic acid	HMDB0000761	2.310	Down	Up
36	Neg	19.99	H_3_O_4_P	194.9457 (2M − H)	160.8408, 96.9583	Hydrogen phosphate	HMDB0000973	1.539	Up	Down
37	Neg	14.92	C_20_H_32_O_3_	639.4633 (2M − H)	319.2279, 179.1067, 320.2313	12R-HETE	—	1.407	Down	Up
38	Neg	14.79	C_22_H_32_O_3_	379.2047 (M + Cl)	343.2279, 344.2314, 205.1226, 281.2274	16(17)-Epoxy-4Z, 7Z, 10Z, 13Z, 19Z-docosapentaenoic acid	HMDB0013621	1.978	Down	Up
39	Neg	14.79	C_22_H_32_O_3_	325.2176 (M − H_2_O − H)	163.1116, 149.0958	17-HDoHE	HMDB0010213	2.644	Down	Up
40	Neg	13.88	C_19_H_28_O_2_	307.2048 (M + F)	271.2279, 272.2313, 225.2218	Androstanedione	HMDB0000899	8.138	Up	Down
41	Neg	11.90	C_19_H_30_O_5_S	391.1533 (M − 2H + Na)	96.968, 78.9574, 96.9589, 293.1759	Androsterone sulfate	HMDB0002759	5.803	Up	Down
42	Neg	14.93	C_23_H_32_O_6_	439.1873 (M + Cl)	96.968, 96.9584, 314.024, 118.8982	Hydrocortisone acetate	HMDB0014879	3.302	Up	Down
43	Neg	13.44	C_18_H_32_O_2_	279.2329 (M − H)	237.2219, 238.2252	Linoleic acid	HMDB0000673	1.791	Down	Up
44	Neg	16.42	C_20_H_32_O_5_	371.2229 (M + F)	217.1225, 195.1018, 163.1115, 149.0962	Lipoxin A4	HMDB0004385	1.347	Up	Down
45	Neg	14.78	C_48_H_84_NO_10_P	864.5755 (M − H)	343.2278	PS (20 : 0/22 : 5(4Z, 7Z, 10Z, 13Z, 16Z))	HMDB0112535	0.000	Down	Up
46	Neg	15.86	C_48_H_90_NO_10_P	870.6232 (M − H)	347.2589, 207.1382, 283.2643, 329.2485, 508.3403, 348.2623	PS (20 : 2(11Z, 14Z)/22 : 0)	HMDB0112585	0.919	Down	Up
47	Neg	17.43	C_50_H_90_NO_10_P	876.6099 (M − H_2_O − H)	339.2328, 340.2361, 163.1115, 303.233, 815.5432, 304.2362	PS (22 : 0/22 : 4(7Z, 10Z, 13Z, 16Z))	HMDB0112730	2.213	Down	Up
48	Neg	14.41	C_50_H_88_NO_10_P	933.6308 (M − H + CH_3_CN)	438.2989, 439.3022, 140.0104, 377.2462, 78.9574	PS (22 : 0/22 : 5(4Z, 7Z, 10Z, 13Z, 16Z))	HMDB0112731	2.731	Down	Up
49	Neg	15.09	C_50_H_88_NO_10_P	892.6074 (M − H)	345.2434, 207.1382, 327.2331, 307.2646, 532.3407, 346.2474	PS (22 : 0/22 : 5(7Z, 10Z, 13Z, 16Z, 19Z))	HMDB0112732	0.672	Down	Up
50	Neg	12.66	C_11_H_22_O_2_	185.1538 (M − H)	61.9867, 141.1272, 116.9271	Undecanoic acid	HMDB0000947	2.160	Up	Down
51	Neg	6.13	C_5_H_11_O_8_P	228.9968 (M − H)	149.0595, 79.9557	Xylulose 5-phosphate	HMDB0000868	6.375	Up	Down
52	Neg	12.71	C_15_H_21_N_5_O_13_P_2_	576.0356 (M + Cl)	159.8587, 157.8619, 540.059	Cyclic ADP-ribose	HMDB0249529	9.809	Up	Down

**Table 4:** Metabolic pathway analysis based on MetaboAnalyst.

No.	Pathway name	Total	Hits	Raw *P*	−Log(*P*)	Impact
1	Linoleic acid metabolism	5	1	0.1109	0.9553	1.0000
2	D-Glutamine and D-glutamate metabolism	6	1	0.1316	0.8809	0.5000
3	Alanine, aspartate, and glutamate metabolism	28	1	0.4848	0.3145	0.1971
4	Selenocompound metabolism	20	1	0.3764	0.4243	0.1591
5	Arginine biosynthesis	14	2	0.0399	1.3986	0.1168
6	Histidine metabolism	16	2	0.0512	1.2910	0.0902
7	Arginine and proline metabolism	38	1	0.5947	0.2257	0.0860
8	Purine metabolism	66	4	0.0631	1.2003	0.0826
9	Pentose and glucuronate interconversions	18	1	0.3461	0.4608	0.0781
10	Sphingolipid metabolism	21	1	0.3911	0.4077	0.0751
11	Pentose phosphate pathway	21	1	0.3911	0.4077	0.0625
12	Beta-alanine metabolism	21	1	0.3911	0.4077	0.0559
13	Primary bile acid biosynthesis	46	2	0.2894	0.5386	0.0390
14	Steroid hormone biosynthesis	77	2	0.5418	0.2662	0.0219
15	Folate biosynthesis	27	1	0.4723	0.3258	0.0203
16	Glutathione metabolism	28	1	0.4848	0.3145	0.0197
17	Valine, leucine, and isoleucine degradation	40	1	0.6137	0.2120	0.0108

## Data Availability

The original data of the RNA-seq analysis has been uploaded to NCBI, https://www.ncbi.nlm.nih.gov/sra/PRJNA801895, which can be downloaded. The raw data used to support the findings of this study are available from the corresponding authors upon request.

## Supplementary Materials

Supplementary Materials Table S1: The contents of the 3 compounds in the RS extract used in this study (x, *n* = 3, %). Figure S1: The chromatography of determination of 3 components in the RS extract by HPLC. (a) Samples at 225 nm and (b) mix standards at 225 nm (1, glucoraphanin; 2, sinapine thiocyanate; 3, sulforaphene).
