# Supplementary material for: Decompression Mechanism of Radish Seed in Prehypertension Rats through Integration of Transcriptomics and Metabolomics Methods
Source: Evid Based Complement Alternat Med. 2023 Jan 31;2023:2139634. doi: 10.1155/2023/2139634 (PMC9904934; doi:10.1155/2023/2139634)
Supplement: Supplementary Materials — Table S1: The contents of the 3 compounds in the RS extract used in this study (x, n = 3, %). Figure S1: The chromatography of determination of 3 components in the RS extract by HPLC. (a) Samples at 225 nm and (b) mix standards at 225 nm (1, glucoraphanin; 2, sinapine thiocyanate; 3, sulforaphene). [file 2139634.f1.doc]

**Supplementary Materials**

Table S1: The contents of the 3 compounds in RS extract used in this study (
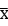
, n=3, %)

| sinapine thiocyanate | glucoraphanin | sulforaphene |
| --- | --- | --- |
| 0.84 | 25.73 | 7.45 |


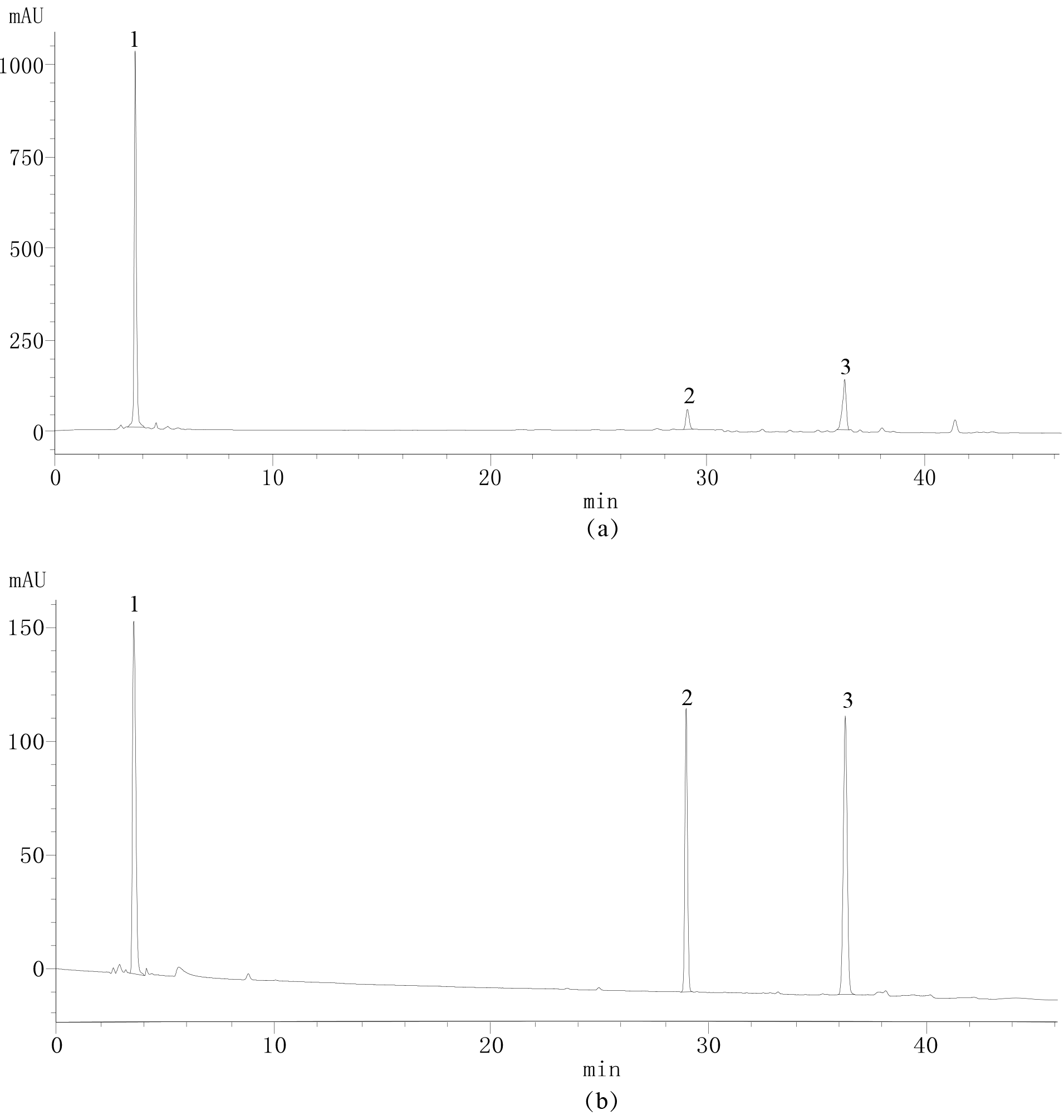


Figure S1: The chromatography of determination of 3 components in RS extract by HPLC. (a)samples at 225 nm, (b)mix standards at 225 nm. (1. glucoraphanin, 2. sinapine thiocyanate, 3. sulforaphene)
